# P2Y_6_ receptor inhibition perturbs CCL2-evoked signalling in human monocytic and peripheral blood mononuclear cells

**DOI:** 10.1242/jcs.159012

**Published:** 2014-11-15

**Authors:** Hinnah Campwala, Darren W. Sexton, David C. Crossman, Samuel J. Fountain

**Affiliations:** 1 School of Biological Sciences, University of East Anglia, Norwich Research Park, Norwich NR4 7TJ, UK; 2 Norwich Medical School, University of East Anglia, Norwich Research Park, Norwich NR4 7TJ, UK

**Keywords:** P2Y_6_, Chemokine, Monocyte, Purinergic signalling

## Abstract

The chemokine CCL2 serves to target circulating monocytes and other leukocytes to tissue during innate immune responses, and modulates the progression of chronic inflammatory disease through activation of the receptor CCR2. Here, we show that co-activation of the P2Y_6_ purinergic receptor (encoded by *P2RY6*) occurs when THP-1 cells and human peripheral blood mononuclear cells sense CCL2 through CCR2. Furthermore, P2Y_6_ receptor activation accounts for ∼80% of the intracellular Ca^2+^ signal evoked by CCL2. Scavenging extracellular nucleotides with apyrase caused a fourfold reduction in THP-1 sensitivity to CCL2, whereas inhibition of CD39-like ectonucleotidases potentiated CCL2-evoked Ca^2+^ responses. Pharmacological inhibition of P2Y_6_ impaired CCL2-evoked Ca^2+^ signalling and chemotaxis in peripheral blood mononuclear cells and THP-1 cells. Furthermore, stable P2Y_6_ receptor knockdown (of twofold) in THP-1 cells impaired CCL2-evoked Ca^2+^ signalling, chemotaxis and adhesion to TNFα-treated HUVECs. We demonstrate that THP-1 cells rapidly secrete ATP during signalling downstream of the CCL2–CCR2 axis and suggest this might act as a mechanism for P2Y_6_ receptor co-activation following CCL2 activation of the CCR2 receptor. The discovery that P2Y_6_ receptor mediates leukocyte responsiveness to CCL2 represents a new mechanism by which to modulate CCL2 signals.

## INTRODUCTION

Chemokines are small secreted peptides that are crucial for the homeostasis of the immune system and its activation during immunity and chronic inflammatory disease. Chemokines operate through activation of G-protein-coupled receptors expressed on the target cells, including leukocytes, directing cells during migration across microanatomical barriers and during interstitial migration (Schabath et al., 2006; Tacke et al., 2007; Griffin et al., 2010; Weninger et al., 2014). They can be subclassified into CC, XC, CXC and CX_3_C chemokines based on the intramolecular configuration and number of disulphide bonds. Chemokines that act in a homeostatic capacity (e.g. CCL19, CCL21 and CXCL13) are secreted constitutively and operate during immune surveillance and tissue maintenance, whereas inflammatory chemokines (e.g. CCL2, CCL5 and CX_3_CL1) are produced by tissue *de novo* in response to tissue damage, infection and inflammation (Cipollone et al., 2001; Namiki et al., 2002; Luther et al., 2002; Spoettl et al., 2006; Serbina and Pamer, 2006). In addition to its inflammatory role, CCL2 (also known as monocyte chemoattractant protein 1, MCP-1) also participates in the egress of monocytes from bone marrow during homeostasis and in the absence of inflammation (Engel et al., 2008). Signalling by inflammatory chemokines is associated with the early onset and progression of several chronic inflammatory diseases, including atherosclerosis, rheumatoid arthritis, diabetes and obesity, where pronounced tissue leukocyte infiltration is a hallmark (Herder et al., 2006; Zernecke and Weber, 2010; O'Boyle et al., 2012). Several small-molecule inhibitors of chemokine receptors are in early and clinical development as therapies (Bachelerie et al., 2014).

Recruitment of circulating monocytes to the arterial wall is an important step in the onset and early progression of atherosclerotic lesions. Animal models of atherosclerosis have demonstrated that signalling by CCL2 and its cognate receptor CCR2 contributes significantly to the magnitude of monocyte/macrophage infiltrate and size of atherosclerotic lesion (Boring et al., 1998; Gu et al., 1998; Veillard et al., 2005; Lutgens et al., 2005). Classical CD14^+^/CD16^−^ blood monocytes highly express CCR2 (Weber et al., 2000), compared to lower expression in CD14^+^/CD16^+^ monocyte subtypes. CCL2 is presented on the cell surface of inflamed endothelium and participates in monocyte recruitment by stimulating integrin-dependent firm adhesion and transmigration of monocytes to the subendothelial space (Wang et al., 1995; Ashida et al., 2001; Maus et al., 2002). Despite the importance of signalling downstream of the CCL2–CCR2 axis, the signal transduction mechanism involved in generating and regulating CCL2-stimulated signals in monocytes remains poorly delineated. CCR2 antagonists have shown varying therapeutic efficacy in clinical trials, and alternative routes to regulating CCL2 activity might prove attractive for future therapeutic strategies (Struthers and Pasternak, 2010).

Leukocytes express a diverse repertoire of receptors for extracellular signalling purines and pyrimidines. Purinergic receptors include P2X (P2X_1–7_), which are ionotropic receptors for ATP (North, 2002), and P2Y (P2Y_1_, P2Y_2_, P2Y_4_, P2Y_6_, P2Y_11_, P2Y_12_, P2Y_13_ and P2Y_14_), which are metabotropic receptors that are activated by ATP, ADP, UTP, UDP or UDP-glucose depending on subtype (von Kügelgen, 2006). Secreted purines and pyrimidines can act in an autocrine and paracrine fashion to relay signals through multiple purinergic receptors expressed at the cell surface. The amount of secreted nucleotide in the pericellular space is tightly regulated by a family of ectonucleotide triphosphate diphosphohydrolases (E-NTPDases) that includes CD39 (also known as ENTPD1), and serve to terminate purinergic signalling through hydrolysis of nucleotide triphosphates and diphosphates to monophosphate forms (Dwyer et al., 2007). We recently identified that THP-1 cells can secrete ATP in response to chemical cues, and that this signalling contributes to constitutive Ca^2+^ homeostasis (Sivaramakrishnan et al., 2012). In neutrophils, ATP is secreted in response to sensing f-Met-Leu-Phe (fMLP) and that this contributes to chemotaxis (Chen et al., 2006).

Here, we investigate the interaction between the purinergic signalling system and the functionality of CCL2 in THP-1 cells and human peripheral blood mononuclear cells (PBMCs). We have shown that P2Y6 activity regulates the magnitude of Ca^2+^ response evoked by signalling downstream of the CCL2–CCR2 axis and plays an important role in CCL2-evoked cellular responses in THP-1 monocytic cells and PBMCs. We find that the majority of Ca^2+^ signal generated following CCR2 activation is via P2Y_6_. We suggest P2Y_6_ co-activation may occur following CCL2 sensing in THP-1 cells and PBMCs via ATP secretion. Antagonism of P2Y_6_ or gene silencing substantially attenuates CCL2-evoked Ca^2+^ signals, chemotaxis towards CCL2 and adhesion of CCL2-primed THP-1 cells to TNFα-treated human umbilical vein endothelial cells (HUVECs). We identify P2Y_6_ as a new and potent modulator of CCL2-dependent signalling in human monocytic and peripheral blood mononuclear cells.

## RESULTS

### CCL2-receptor-mediated Ca^2+^ signalling is required for chemotaxis

**Fig. 1.**
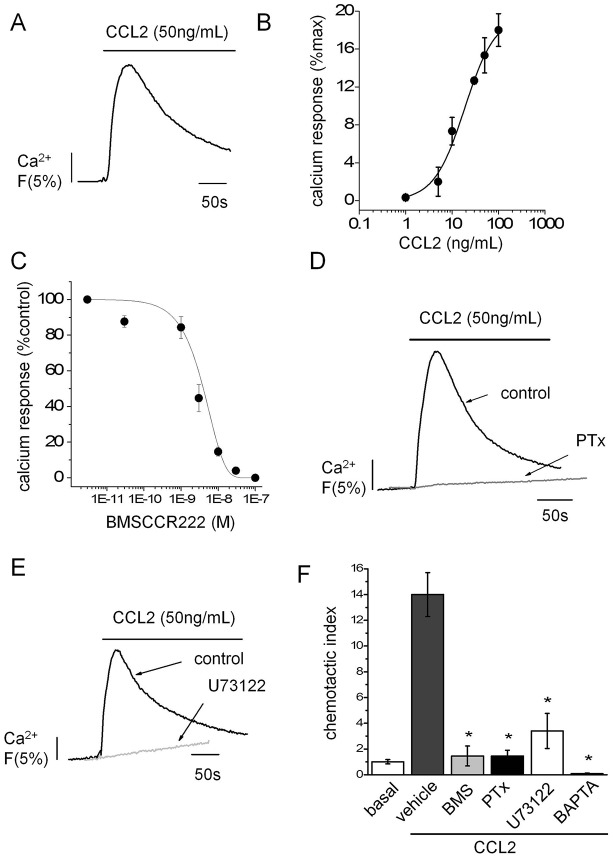
**CCL2 receptor mediated Ca^2+^ signalling is required for chemotaxis.** (A) Representative Ca^2+^ transient evoked by CCL2 in Fluo-4-loaded THP-1 cells. (B) Relationship between peak Ca^2+^ response and CCL2 concentration in THP-1 cells (*n* = 3). (C) Effect of the selective CCR2 antagonist BMSCCR222 on peak Ca^2+^ response to 50 ng/ml CCL2 expressed as a percentage of control (no antagonists) in THP-1 cells (*n* = 3). (D) Representative trace showing effect of PTx (100 ng/ml) on CCL2-evoked Ca^2+^ responses in THP-1 cells (*n* = 3). (E) Representative trace showing effect of PLC inhibition with U73122 (5 µM) on CCL2-evoked Ca^2+^ responses in THP-1 cells (*n* = 4). (F) Transwell assays showing the effect of PTx (100 ng/ml), BMSCCR222 (BMS, 100 nM), U73122 (5 µM) and BAPTA-AM (100 µM) (all *n* = 3) on THP-1 migration towards CCL2 (50 ng/ml lower chamber, 2 h). Fluo-4 signals are normalised to the maximum Ca^2+^ response elicited by 40 µM digitonin. *P<0.01 (ANOVA) versus vehicle control. Data are presented as mean±s.e.m. All antagonists were pre-incubated for 30 min prior to agonist challenge.

CCL2-evoked intracellular Ca^2+^ signals in THP-1 cells with a half-maximal concentration of 15±3 ng/ml (mean±s.e.m., *n* = 3; Fig. 1A,B). Ca^2+^ signals evoked by CCL2 were abolished by BMSCCR222 (IC_50_ = 2.9±0.3 nM; *n* = 3) demonstrating that CCL2-evoked signals are mediated through activation of CCR2 (Fig. 1C). CCL2-evoked Ca^2+^ signals were ablated by *Bordetella pertussis* toxin (PTx; 100 ng/ml; *n* = 3) and U 73122 (5 µM; *n* = 4) suggesting that the subsequent signal transduction events involved in Ca^2+^ signal development following CCR2 activation are dependent on Gαi-type heterotrimeric G proteins and phospholipase C (PLC) (Fig. 1D,E). Furthermore, THP-1 cell migration towards CCL2 was inhibited by BMSCCR222, PTx and following chemical chelation of cytoplasmic Ca^2+^ by BAPTA AM (Fig. 1F). These data suggest that CCR2-mediated Ca^2+^ signals are essential for THP-1 cell migration towards CCL2.

### Scavenging extracellular signalling nucleotides suppressed CCL2-evoked Ca^2+^ signalling and cell migration

**Fig. 2.**
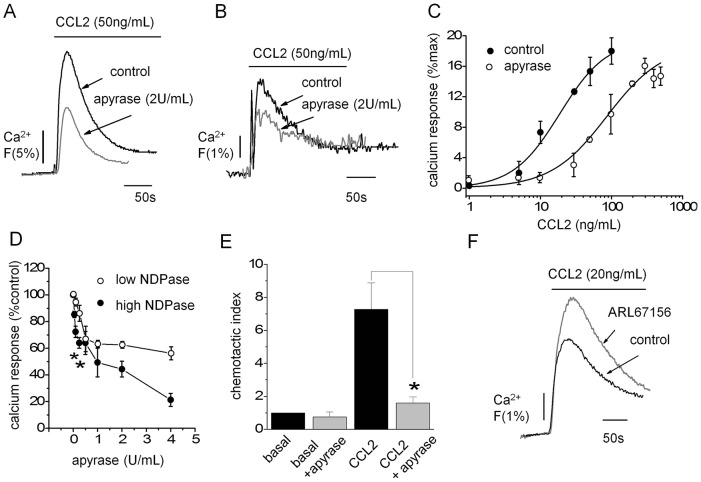
**Effect of modulating extracellular nucleotides on THP-1 and human PBMC responses to CCL2.** Representative traces showing effect of the nucleotide-scavenging enzyme apyrase on the CCL2-evoked Ca^2+^ signal in THP-1 cells (A) and human PBMCs (B). (C) Apyrase (2 U/ml) induced a rightwards parallel shift in the CCL2 concentration–response relationship for peak Ca^2+^ responses in THP-1 cells (*n* = 3). (D) Effect of apyrase enzymes with equal NTPase activity but high or low NDPase activity on peak Ca^2+^ responses evoked by CCL2 (50 ng/ml) expressed as a percentage of the response in the absence of enzyme (*n* = 3; *P<0.05, Students *t*-test). Enzyme units refers to NTPase activity. (E) Apyrase (2 U/ml) suppresses THP-1 chemotaxis towards CCL2 (50 ng/ml lower chamber, 2 h) in transwell migration assays (*n* = 5; *P<0.01, ANOVA). (F) Representative trace showing that the response to submaximal concentrations of CCL2 is potentiated following E-NTPDase inhibition (ARL67156, 100 µM; *n* = 9). Fluo-4 signals are normalised to maximum Ca^2+^ response elicited by 40 µM digitonin. Data are presented as mean±s.e.m. All antagonists were pre-incubated for 30 min prior to agonist challenge.

To determine the role of purinergic signalling in regulating CCL2-evoked Ca^2+^ responses, we initially sought to investigate the effect of modulating endogenous extracellular nucleotides through application of CD39-like apyrase and through pharmacological inhibition of E-NTPDases. Apyrase (2 U/ml) attenuated CCL2-evoked Ca^2+^ signalling in both THP-1 monocytic cells and human PBMCs (Fig. 2A,B), with peak responses suppressed by 59%±5 (*n* = 6) and 47%±3 (mean±s.e.m., *n* = 3), respectively. In THP-1 cells, the inhibition was marked by a fourfold parallel rightward shift in the CCL2 concentration–response curve for Ca^2+^ responses, indicating reduced efficacy of CCL2 signalling during extracellular nucleotide scavenging (Fig. 2C). To investigate the dependency of the response on extracellular nucleotide triphosphates (NTPs; ATP, UTP) versus nucleotide diphosphates (NDPs; ADP, UDP) we used two different types of apyrases, one with a low NTPase:NDPase ratio and one with a high NTPase:NDPase ratio. Both apyrase isoforms were applied to give equal NTPDase activity, but the NDPase activity would either be high or low dependent on the isoform. We observed that enzymes with high NDPase activity produced greater inhibition of CCL2 evoked Ca^2+^ signals (Fig. 2D). At 4 U/ml NTPase activity, apyrase with high NDPase activity caused ∼twofold more inhibition than low-NDPase activity apyrase (Fig. 2D). These data suggest that the availability of extracellular NDPs is a strong determinant of CCL2 signalling efficiency. Apyrase treatment also suppressed THP-1 migration towards CCL2 (Fig. 2E). Apyrase treatment had no effect on the cell surface expression of CCR2 (supplementary material Fig. S1). In reciprocal experiments, Ca^2+^ signals to sub-maximal concentrations of CCL2 could be potentiated following E-NTPDase inhibition (ARL 67156, 100 µM; 19%±3; *n* = 9) (Fig. 2F). The data suggest that the bioavailability of extracellular nucleotides is a major determinant of CCL2 signalling efficacy.

### P2Y_6_ activation amplifies CCL2-evoked Ca^2+^ signalling in THP-1 cells

**Fig. 3.**
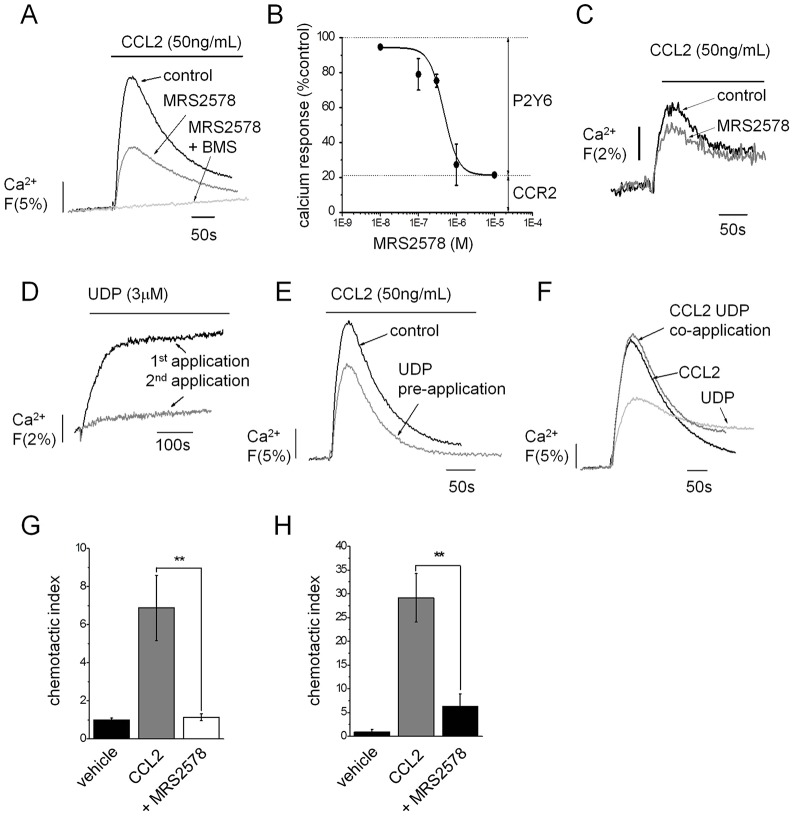
**P2Y_6_ receptor antagonism and desensitization suppresses Ca^2+^ signalling downstream of the CCL2–CCR2 axis.** (A) Representative traces demonstrating the dependency of CCR2 and P2Y_6_ for CCL2-evoked Ca^2+^ signalling in THP-1 cells. Traces shown are for responses in the absence of antagonist (control), following selective P2Y_6_ inhibition (MRS2578, 1 µM) and following inhibition of P2Y_6_ and CCR2 receptors [MRS2578 + BMSCCR222 (BMS), 100 nM]. (B) Concentration–response relationship showing inhibitory action of MRS2578 on peak Ca^2+^ responses to CCL2 (50 ng/ml). The MRS2578-resistant component of ∼20% accounts for CCR2 activity (*n* = 3). (C) Representative trace showing inhibition of CCL2-evoked Ca^2+^ signalling in human PBMCs following P2Y_6_ inhibition (MRS2578, 1 µM) (*n* = 3). (D) Representative traces showing desensitization of UDP (3 µM)-evoked Ca^2+^ response in subsequent applications 10 min apart. (E) Representative trace showing effect of UDP (3 µM) pre-application (as in D) on CCL2 (50 ng/ml)-evoked Ca^2+^ response in THP-1 cells. (F) Representative traces showing lack of synergy between CCL2 (50 ng/ml) and UDP (30 µM) as revealed by co-application experiments. P2Y_6_ receptor inhibition (MRS2578, 1 µM) inhibition of THP-1 cell (G) and PBMC (H) chemotaxis towards CCL2 (50 ng/ml lower chamber, 2 h) in transwell migration assays (*n* = 3–4). Fluo-4 signals are normalised to the maximum Ca^2+^ response elicited by 40 µM digitonin. **P<0.01 (ANOVA). Data are presented as mean±s.e.m. All antagonists were pre-incubated for 30 min prior to agonist challenge.

As apyrase with high NDPase activity produced greater inhibition of CCL2-evoked Ca^2+^ responses compared to apyrase with low NDPase activity (Fig. 2D), we investigated the role of UDP/ADP-activated P2Y receptors in facilitating CCL2 signalling. Candidate receptors expressed in THP-1 cells and primary human monocytes (data not shown) included P2Y_1_ (ADP), P2Y_6_ (ADP, UDP), P2Y_11_ (ADP), P2Y_12_ (ADP) and P2Y_13_ (ADP). Selective antagonism of P2Y_1_, P2Y_11_, P2Y_12_ or P2Y_13_ with MRS 2179, NF 340, AR-C 66096 or MRS 2211, respectively, had no effect on CCL2-generated Ca^2+^ signals or THP-1 migration towards CCL2 (data not shown). In contrast, the selective P2Y_6_ receptor antagonist MRS 2578 inhibited CCL2-evoked Ca^2+^ signals in THP-1 cells (IC_50_ = 418±68 nM, mean±s.e.m., *n* = 3) (Fig. 3A,B) and in PBMCs (Fig. 3C). MRS2578 treatment had no effect on the cell surface expression of CCR2 (supplementary material Fig. S1). P2Y_6_ inhibition revealed a persistent component of the CCL2-evoked Ca^2+^ signal that was ∼20% of maximum (Fig. 3B). The resistant component was abolished by selective CCR2 antagonism with BMSCCR222 (Fig. 3A). These data suggest that engagement of P2Y_6_ accounts for ∼80% of the maximal Ca^2+^ response to CCL2 and that activation of the CCR2 receptor alone, although indispensible, generates only 20% of the maximal response. Ca^2+^ responses to UDP were unaffected by CCR2 inhibition (data not shown) suggesting P2Y_6_ activity does not reciprocate a dependency on CCR2 activity. CCL2-evoked Ca^2+^ responses in PBMCs were also antagonised by MRS2578 (34%±9; *n* = 3 donors, *P*<0.05). Further evidence for CCL2-dependency upon P2Y_6_ activity was sought by desensitizing P2Y_6_ with supramaximal concentrations of ADP or UDP agonist prior to CCL2 application. UDP elicited a modest sustained Ca^2+^ response (Fig. 3D), characteristic of the slow desensitization kinetics of P2Y_6_. Application of 3 µM UDP or ADP desensitized subsequent nucleotide responses by 68%±10 and 40%±8 (*n* = 3–5), respectively (Fig. 3D). CCL2 responses were attenuated by UDP (25%±4; *n* = 5) and ADP (27%±5; *n* = 3) when applying the same desensitization protocol (Fig. 3E). No additional response over CCL2 alone was observed when CCL2 and UDP were co-applied (Fig. 3F). These data suggest P2Y_6_ serves to amplify the CCL2 response through co-activation following CCL2 sensing in monocytes. Our observations in Ca^2+^ signal experiments were mirrored in functional assays whereby P2Y_6_ inhibition reduced migration to CCL2 in both THP-1 cells (82%±2; *n* = 4) (Fig. 3G) and PBMCs (82%±5; *n* = 3 donors) (Fig. 3H).

### P2Y_6_ engagement is not required for fMLP signalling

**Fig. 4.**
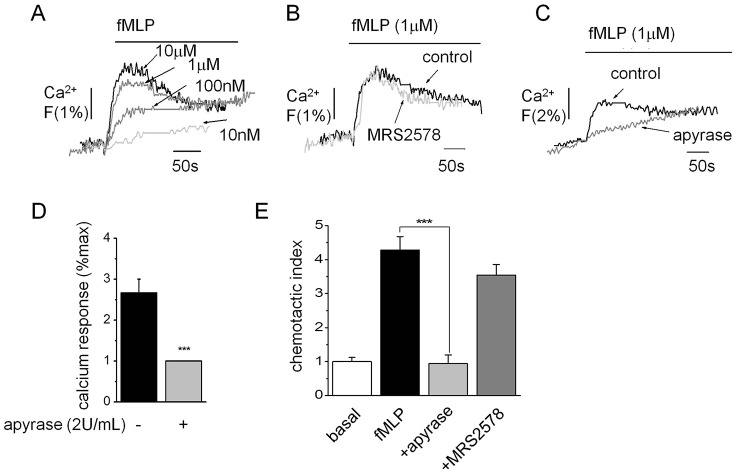
**The fMLP-evoked Ca^2+^ response and chemotaxis are not dependent on P2Y_6_ receptor activity in THP-1 cells.** (A) Representative Ca^2+^ transients evoked by various concentrations of fMLP. (B) Representative paired trace showing the lack of effect on fMLP (1 µM)-evoked Ca^2+^ responses following P2Y_6_ receptor inhibition (MRS2578, 1 µM). (C) Trace showing inhibitory action of apyrase (2 U/ml) on the magnitude of response evoked by fMLP (1 µM) with average data shown in D. Apyrase (2 U/ml), but not MRS2578 (1 µM), inhibits THP-1 cell chemotaxis towards fMLP (1 µM lower chamber, 2 h) in transwell migration assays (*n* = 4). Fluo-4 signals are normalised to the maximum Ca^2+^ response elicited by 40 µM digitonin. ***P<0.01 (ANOVA). Data are presented as mean±s.e.m. All antagonists were pre-incubated for 30 min prior to agonist challenge.

To rule out a generalized role of P2Y_6_ engagement in amplifying Ca^2+^ responses to other chemotactic peptides, we investigated the effect of P2Y_6_ inhibition on Ca^2+^ responses and cell migration mediated by fMLP. Nanomolar fMLP elicited concentration-dependent Ca^2+^ responses in THP-1 cells (Fig. 4A). Responses were insensitive to maximal concentrations of MRS2578 (Fig. 4B), although they were attenuated by apyrase (61%±6, mean±s.e.m., *n* = 3) (Fig. 4C,D). Cell migration towards fMLP was also insensitive to MRS2578 but inhibited by apyrase (Fig. 4E). These data exclude a role of P2Y_6_ in fMLP-dependent signalling in monocytic cells but highlight a potential role for other P2 receptor subtypes.

### P2Y_6_-knockdown THP-1 cells display impaired CCL2-evoked migration and adhesion to TNFα-treated HUVECs

**Fig. 5.**
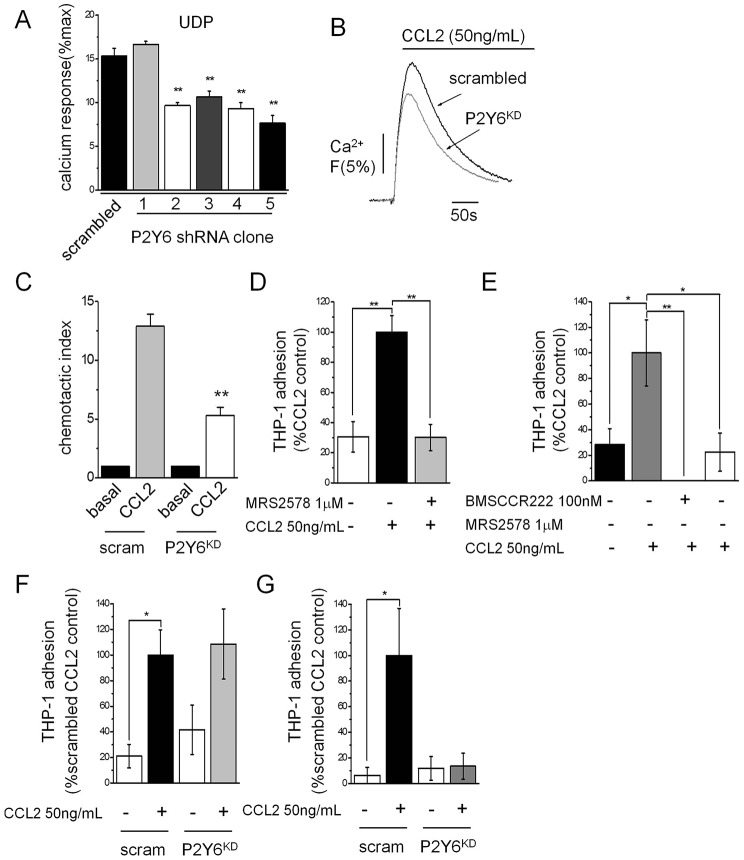
**P2Y_6_-knockdown THP-1 cells have impaired CCL2-evoked Ca^2+^ signalling, chemotaxis and adhesion to TNFα-treated HUVECs.** (A) Comparison of UDP (30 µM)-evoked Ca^2+^ responses in THP-1 stable lines expressing scrambled shRNA or P2Y_6_-targeted shRNA derived from 5 different clones (clone 1–5) (*n* = 3). Functional knockdown of UDP responses are revealed in clones 2–5. (B) Representative traces showing the attenuated Ca^2+^ response to CCL2 in P2Y_6_-knockdown (P2Y_6_^KD^) cells. (C) Impaired chemotactic response to CCL2 (50 ng/ml lower chamber, 2 h) observed in transwell migration assays for P2Y_6_^KD^ cells versus scrambled THP-1 cells (*n* = 4). (D) CCL2 priming (50 ng/ml, 45 min) of THP-1 cells stimulated adhesion to non-inflamed HUVECs. P2Y_6_ inhibition (MRS2578, 1 mM) abolished CCL2-evoked adhesion (*n* = 20). (E) In inflamed HUVECs (TNFα, 10 ng/ml, 5 h), CCL2 priming (50 ng/ml, 45 min) of THP-1 cells also stimulated adhesion that could be blocked by pre-treatment with either BMSCCR222 (100 nM) or MRS2578 (1 µM) (E) (*n* = 8 for both). (F) P2Y_6_ knockdown causes variable adhesion of CCL2-primed THP-1 cells to TNFα-treated HUVECs as compared for scrambled (scram) and P2Y_6_-knockdown lines (*n* = 6). (G) CCL2-primed P2Y_6_-knockdown THP-1 cells display impaired adhesion to inflamed HUVECs (TNFα, 10 ng/ml, 5 h) versus scrambled counterparts (*n* = 8). P2Y_6_^KD^ refers to clone 5 (as shown in A) throughout. Fluo-4 signals are normalised to the maximum Ca^2+^ response elicited by 40 µM digitonin. **P<0.01, *P<0.05 (ANOVA). Data are presented as mean±s.e.m. All antagonists were pre-incubated for 30 min prior to agonist challenge.

To support our pharmacological data, we generated stable P2Y_6_-knockdown THP-1 lines through lentivirus-mediated shRNA delivery. From a screen of five shRNA clones, the best performing clone achieved a 50%±5 (mean±s.e.m., *n* = 3) reduction in the functional Ca^2+^ response to UDP compared to scrambled shRNA-expressing cells (Fig. 5A). Clone 5 (Fig. 5A) showed a twofold knockdown of P2Y_6_ (*P2RY6*) mRNA transcripts and was used in all subsequent experiments. P2Y_6_ knockdown did not alter the level of CCR2 mRNA transcripts (data not shown), and had no effect on the cell surface expression of CCR2 (supplementary material Fig. S1). P2Y_6_ protein levels were not probed owing to lack of specific P2Y_6_ antibodies (Yu and Hill, 2013). P2Y_6_ knockdown THP-1 cells displayed a marked reduction (20%±1; *n* = *3*) in the peak Ca^2+^ response to CCL2 versus scrambled shRNA counterparts (Fig. 5B). P2Y_6_-knockdown cells also displayed reduced migration towards CCL2 (Fig. 5C). CCL2 participates in early monocyte recruitment events such as adhesion to endothelium (Gerszten et al., 1999; Maus et al., 2002; Hiraoka et al., 2004). To this end, we sought to test the impact of P2Y_6_ receptor knockdown on CCL2-stimulated THP-1 adhesion to vascular endothelium. CCL2 priming of THP-1 cells enhanced adhesion to both non-inflamed and inflamed (TNFα; 10 ng/ml, 5 h) HUVEC monolayers (Fig. 5D–F). Adhesion stimulated by CCL2 could be abolished by CCR2 antagonism with BMSCCR222 (Fig. 5D,E). P2Y_6_ knockdown had no significant effect on THP-1 adhesion to non-inflamed endothelium (Fig. 5F) but caused a substantial inhibition of adhesion to inflamed cells (Fig. 5G). Adhesion to TNFα-treated HUVECs could also be inhibited by MRS2578 (78%±15; *n* = 8) (Fig. 5F). Taken together, these data support a role of P2Y_6_ in monocyte function associated with inflammation.

### CCL2-stimulated ATP secretion in THP-1 cells

**Fig. 6.**
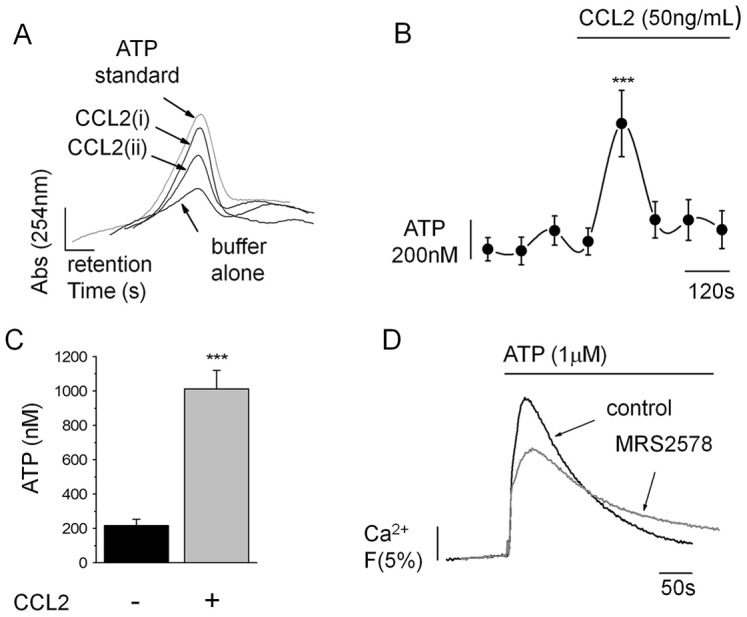
**CCL2-coupled ATP secretion.** (A) CCL2-stimulated ATP secretion from THP-1 cells detected by HPLC. Representative traces show HPLC analysis of THP-1-conditioned buffer following no stimulation (buffer alone) or collected immediately after (CCL2 i) or 2 min after (CCL2 ii) CCL2 (50 ng/ml) stimulation. Peak retention and amplitude are shown in comparison to a 1 µM ATP standard. (B) ATP content in THP-1-conditioned buffer collected before and after CCL2 (50 ng/ml) stimulation as quantified by luciferase bioluminescence (*n* = 7 for each time point). (C) Mean peak ATP secreted following CCL2 stimulation quantified by luciferase bioluminescence (*n* = 7). (D) Representative traces showing the suppression of the Ca^2+^ response evoked by 1 µM exogenous ATP following P2Y_6_ receptor inhibition (MRS2578, 1 µM). Fluo-4 signals are normalised to the maximum Ca^2+^ response elicited by 40 µM digitonin. ***P<0.01 (ANOVA). Data are presented as mean±s.e.m. All antagonists were pre-incubated for 30 min prior to agonist challenge.

We have previously demonstrated that THP-1 cells can secrete ATP in response to chemical cues (Sivaramakrishnan et al., 2012; Campwala and Fountain, 2013). It is, therefore, plausible that ATP secreted in response to CCL2 could liberate ADP required to activate P2Y_6_ through cell surface E-NTPDase activity. We undertook experiments using reverse-phase high-pressure liquid chromatography (HPLC) to determine the species of nucleotide secreted from THP-1 cells following CCL2 challenge, to gain evidence for release of ADP or UDP P2Y_6_ ligands, or UTP and ATP precursor molecules. Our HPLC methodology allowed detection of ATP, UTP, ADP and UDP standards with a detection threshold close to 1 µM. Despite this, ATP was the only nucleotide that showed increased secretion from THP-1 cells challenged with CCL2. ADP was rarely detected in substantial amounts. Extracellular ATP increased in a time-dependent fashion following CCL2 challenge (Fig. 6A). Evidence for CCL2-evoked ATP secretion was supported by luciferin-luciferase assays. In these experiments, CCL2 evoked a transient elevation in extracellular ATP with a peak amplitude of 1013±108 nM (mean±s.e.m., *n* = 7) (Fig. 6B,C). Ca^2+^ responses in THP-1 cells evoked by 1 µM exogenous ATP were antagonized by P2Y_6_ inhibition (31%±1, *n* = 3) (Fig. 6D) demonstrating the capacity of secreted ATP, at the levels detected, to activate P2Y_6_. Without a direct measure of UDP secretion, we cannot exclude the possibility that its secretion is below the detection limits of our HPLC methodology. UDP acted at the human P2Y_6_ receptor stably expressed in 1321N1 cells with a half-maximal concentration of 23±3 nM, compared to 3.4±0.2 mM for ADP (data not shown). We propose that agonist-induced nucleotide secretion is a possible mechanism underlying co-activation of P2Y_6_ following CCR2 activation.

## DISCUSSION

In this study, we have identified a new signalling interaction between the CCR2 receptor (for CCL2) and the P2Y_6_ receptor (for ADP and UDP) in THP-1 monocytic cells and human PBMCs. Our results clearly indicate that, although CCL2-mediated Ca^2+^ responses show an absolute requirement for CCR2, subsequential activation of the P2Y_6_ receptor serves to substantially amplify the Ca^2+^ signal. This interaction between CCR2 and P2Y_6_ receptor activity is essential for THP-1 chemotaxis and adhesion to inflamed vascular endothelium in response to CCL2. The activity of P2Y_6_ appears to differentially regulate the response to chemotactic peptides as inhibition of P2Y_6_ or silencing its expression with shRNAs in THP-1 cells impairs the responses to CCL2, but the responsiveness of these cells to fMLP is independent of P2Y_6_ receptor activity. However, our study does support a role of extracellular signalling nucleotides in controlling Ca^2+^ responses and cellular migration to fMLP, as apyrase impairs both these processes. The data support previous studies showing fMLP-stimulated chemotaxis in human neutrophils is impaired by apyrase treatment (Chen et al., 2006). Our work also supports the hypothesis that CCR2 receptors couple to Gαi-type heterotrimeric G proteins (Chen et al., 2006), and that Ca^2+^-evoked signalling is mediated through PLC activity. Although U73122 cannot discriminate between PLC isoforms, the coupling is likely through interaction of Gβγ subunits with PLCβ isoforms (Park et al., 1992). Indeed, we observed that the Gβγ antagonist gallein inhibits CCL2-stimulated chemotaxis in THP-1 cells (data not shown). What is clear from our study is that CCL2-evoked Ca^2+^ signals are essential for THP-1 chemotaxis towards CCL2 as inhibition of Gαi, PLC and chemical buffering of intracellular Ca^2+^ with BAPTA all substantially attenuate THP-1 cell migration towards CCL2. The activity of phospholipase enzymes and the generation of Ca^2+^ signals are crucial for leukocyte movement (Cathcart, 2009). Indeed, localized Ca^2+^ signalling events at the leading edge of migrating leukocytes is important for the organization of actin and focal complex assembly during the formation of pseudopods (Mishra et al., 2008). In transwell migration assays, we observed that chelating intracellular Ca^2+^ with BAPTA-AM inhibited the level of basal THP-1 migration. Changes in cytoplasmic Ca^2+^ are important for cell polarization and motility during chemotaxis (Wei et al., 2012), but also influence random cell migration where spontaneous Ca^2+^ events are believed to play a role (Lee et al., 2012).

In experiments involving apyrase, we demonstrate that nucleotide scavenging causes an approximate fourfold shift in CCL2 potency during Ca^2+^ signalling. The attenuation is surmounted by higher CCL2 concentrations, but the activation threshold for CCL2 in apyrase-treated THP-1 cells is >50 ng/ml versus 5 ng/ml for untreated cells. Based on our data, apyrase treatment might render monocytic cells non-responsive to the small, but substantially elevated, concentrations of plasma CCL2, as detected in sepsis (Bossink et al., 1995), sarcoidosis (Hashimoto et al., 1998), subclinical atherosclerosis (Deo et al., 2004) and type II diabetes (Piemonti et al., 2009). In reciprocal experiments, increasing the pericellular availability of nucleotide triphosphate and diphosphate by E-NTPDase inhibition with ARL67156 potentiated responses to CCL2. These data clearly demonstrate that the levels of extracellular nucleotides modulate how monocytic cells and PBMCs respond to CCL2 by controlling the detection threshold of CCL2. In an *in vivo* setting, this would suggest CCR2^+^ leukocytes in environments of high E-NTPDase activity would have dampened responses to CCL2. The prototypical E-NTPDase CD39 plays a strategic role in calibrating the magnitude, duration and chemical nature of signalling purines and pyrimidine signals presented to immune cells (Antonioli et al., 2013), and is highly expressed by regulatory T cells that decrease the formation of atherosclerotic lesion (Borsellino et al., 2007; Dinh et al., 2012). The anti-inflammatory activity of CD39 is attributed to limiting extracellular ATP accumulation, which suppresses production and release of pro-inflammatory cytokines, such as interleukin (IL)-1β, IL-6, IL-18 and TNFα from leukocytes (Reutershan et al., 2009; Lévesque et al., 2010). The activity of CD39 expressed by resting vascular endothelium is important for maintaining the antithrombotic low ATP and ADP environment (Packham and Mustard, 2005; Fung et al., 2009). Our study reveals an additional new anti-inflammatory role for CD39-like E-NTPDases by limiting the availability of P2Y_6_ ligand and suppressing signalling downstream of the CCL2–CCR2 axis.

We have demonstrated the ability of human THP-1 cells to secrete ATP in this study and in previous studies (Sivaramakrishnan et al., 2012; Campwala and Fountain, 2013). ATP secretion in response to sensing chemoattractants might be a common mechanism in leukocytes, as human neutrophils secrete ATP in response to fMLP (Chen et al., 2006). In this study, we detected an elevation in ATP of ∼1 µM following exposure to CCL2. Our bulk-phase measurements are likely to underestimate the pericellular concentration of extracellular ATP. Indeed, pericellular measurement of ATP secretion evoked by fMLP in neutrophils reveals levels close to 30 µM (Chen et al., 2006). Our data also supports a role for extracellular nucleotides in fMLP-mediated responses as apyrase impairs fMLP-evoked Ca^2+^ signals and chemotaxis. However, our study does not support a role for P2Y_6_. Chen et al. (Chen et al., 2006) suggest a role for the P2Y_2_ receptor for ATP and/or UTP in supporting fMLP signalling in neutrophils, which do not express P2Y_6_. We show that P2Y_6_ plays a substantial role in amplifying signalling downstream of the CCL2–CCR2 axis and have ruled out the involvement of other P2Y receptors (based on selective antagonism), although we cannot rule out a contribution of P2Y_2_ in amplifying the CCL2 response. Our data suggest that the contribution of non-P2Y_6_ purinergic receptors would be small based on the magnitude of inhibition with MRS2578. In addition, we observed that MRS2578 inhibits CCL2-evoked Ca^2+^ signals to a lesser extent in PBMCs than in THP-1 cells. We suggest that although CCR2 is the cognate receptor for CCL2, the PBMC fraction will contain a mixed population of cells, some of which might not express P2Y_6_ or might express P2Y_6_ to a varied degree compared to in THP-1 cells. Exploring the functional interaction between P2Y_6_ and CCL2 in leukocyte subtypes is therefore an important future work.

Several important questions arise from this study. Activation of CCR2 by CCL2 and N-formyl peptide receptors by fMLP both raise cytoplasmic Ca^2+^ by pertussis-toxin-sensitive mechanisms (Krause et al., 1985), and Ca^2+^ responses and chemotaxis evoked by both are substantially impaired by apyrase. The suggestion here is that fMLP might cause ATP release in human monocytes as in neutrophils (Chen et al., 2006). Despite these similarities, CCL2 responses are highly dependent upon P2Y_6_ receptor activation, although fMLP responses are independent of P2Y_6_ activity. A possible explanation for this might be that CCR2, P2Y_6_ and the ATP release site are in close proximity. This might be an important spatial arrangement as CD39-like activity would mean that secreted ATP would have a very short half-life and would be unlikely to diffuse significantly across the cell surface to target receptors. One would therefore assume the formyl peptide receptors and P2Y_6_ are not in close proximity or that fMLP does not evoke secretion of P2Y_6_ receptor ligand. Localised association of CCR2 and P2Y_6_ might be important for amplifying local external CCL2 signals, enabling cell orientation and migration in chemotactic fields. A further question raised by this study is how CCR2 activation couples to ATP secretion. Several routes have been proposed for ATP release, including lysosome secretion (Sivaramakrishnan et al., 2012), connexin, pannexin and hemichannels (Huang et al., 2007; Kang et al., 2008), and ion channels (Hisadome et al., 2002; Suadicani et al., 2006; Romanov et al., 2008). Understanding how CCR2 activation couples to ATP secretion might also provide targets to suppress CCL2-evoked signals. Our data suggest that targeting P2Y_6_ would impede CCL2-dependent tissue recruitment of monocytes at two keys steps, chiefly monocyte adhesion to inflamed vascular endothelium and monocyte migration. In addition, our data reveal a varying dependency on P2Y_6_ for CCL2-mediated THP-1 adhesion to non-inflamed versus inflamed HUVECs. This is an intriguing observation but is difficult to provide a concise explanation. We suggest that P2Y_6_ receptor engagement serves to amplify the CCL2 signal; however, residual CCR2-dependent signalling can still occur when P2Y_6_ activity is pharmacologically inhibited. This might suggest that the magnitude of CCL2-evoked signalling in the absence of P2Y_6_ is sufficient to allow THP-1 adhesion to non-inflamed, but not inflamed, HUVECs.

## MATERIALS AND METHODS

### Chemicals and reagents

All chemicals were purchased from Sigma-Aldrich (UK) with the exception of BMSCCR222, BAPTA AM, MRS2578, ARL67156, AR-C66096 and *Bordetella pertussis* toxin (PTx; Tocris Bioscience, UK), MRS2179, MRS2211 (Abcam Biochemicals, UK), CCL2, TNFα and Fluo-4 AM (Life Technologies, UK), and Calcein AM (Santa Cruz Biotechnology, Germany). THP-1 and HEK 293T cells were procured from the European Collection of Cell Cultures (ECACC), and HUVECs from Caltag Medsystems (UK). Human 1321N1 P2Y_6_ stable cells were a kind gift from Jens Leipziger (Aarhus University, Denmark). Compounds used did not induce toxicity under assay conditions as determined by a Trypan Blue exclusion assay or lactate dehydrogenase (LDH) release assay.

### Cells

THP-1 cells (human acute monocytic leukaemia) were cultured in RPMI 1640 medium with 2 mM L-glutamine, 10% (v/v) heat-inactivated foetal bovine serum (HI-FBS), and 50 IU/ml penicillin and 50 µg/ml streptomycin. Cultures were maintained between 1×10^5^ and 1×10^6^ cells/ml. Early passage HUVECs were cultured to confluency in endothelium cell growth medium (PromoCell GmbH, Germany). 1321N1 hP2Y_6_ stable cells were transfected as described previously (Communi et al., 1996) with recombinant pcDNA3 plasmid encoding human P2Y_6_ and were grown in DMEM medium supplemented with 10% (v/v) HI-FBS and 0.4 mg/ml G418. All cultures were maintained at 37°C in a humidified 5% CO_2_ incubator.

### Isolation of PBMCs

Peripheral venous blood was collected from healthy human volunteers in sodium citrate solution (1:10 volumes). Blood was diluted 1:1 with Dulbecco's phosphate-buffered saline (dPBS) before being transferred to Accuspin tubes (containing Histopaque 1077; Sigma-Aldrich, UK) for centrifugation at 1000 ***g*** for 10 min. The resultant buffy layer was carefully removed and washed three times with dPBS (1:1 volumes), centrifuging at 250 ***g*** for 10 min in between washes before final collection of PBMCs. Use of blood from healthy volunteers was approved by the Faculty of Medicine and Health Sciences Research Ethics Committee, University of East Anglia (UK).

### Intracellular Ca^2+^ measurements

For intracellular Ca^2+^ measurements with THP-1 cells, 10^6^ THP-1 cells/ml were loaded for 1 h at 37°C with 2 µM Fluo-4 AM in SBS buffer containing: 130 mM NaCl, 5 mM KCl, 1.2 mM MgCl_2_, 1.5 mM CaCl_2_, 8 mM D-glucose, 10 mM HEPES pH 7.4, plus 0.01% (w/v) pluronic acid. Cells were re-suspended at 10^6^ cells/ml in SBS. Fluo-4 AM fluorescence (494 nm excitation; 516 nm emission), was sampled at room temperature at 1-s intervals using a Hitachi F-2000 fluorescence spectrophotometer. Cells in a quartz cuvette were continuously agitated by means of a magnetic stirrer. Maximum fluorescence (*F*_max_) signals were generated by the addition of 40 µM digitonin. Ca^2+^ responses to drugs were expressed as a percentage of *F*_max_. Experiments were performed at room temperature (22–25°C). Intracellular Ca^2+^ measurements were with human PBMCs performed as described above using 2×10^7^ cells/ml loaded in 4 µM Fluo-4 AM.

### Transwell migration assays

Transwell migration assays with THP-1 cells were performed in 24-well plates using polyethylene terephthalate (PET) membrane transwell inserts with 3-µm pores (BD Biosciences, UK). THP-1 cells (10^6^) in RPMI without serum were added to upper chambers, and 50 ng/ml CCL2 or 1 µM fMLP or vehicle were added to lower chambers. Compounds were added to upper wells as appropriate. Cell migration was allowed to progress for 2 h at 37°C in a humidified 5% CO_2_ incubator. Inserts were washed twice in ice-cold dPBS and fixed with −20°C methanol. Upper chambers were swabbed and cells stained with 0.5% (w/v) Crystal Violet. Migrated cells were scored using an Olympus CKX41 inverted microscope equipped with a Leica digital camera. Cell migration was assessed by calculating the chemotactic index (CI) by dividing the number of cells migrating in response to treatment by the number of spontaneously migrated cells. Transwell migration assay with human PBMCs were performed as described above using 1×10^6^ cells/ml in Hanks balanced salt solution containing 2.9 g/l HEPES.

### HUVEC adhesion assay

HUVECs were plated onto wells of a black, clear-bottomed 96-well plate and allowed to form confluent monolayers over 48 h at 37°C in a humidified 5% CO_2_ incubator. HUVEC monolayers were washed once with SBS buffer prior to pre-treatment with TNFα (10 ng/ml) or vehicle (SBS buffer) for 5 h at 37°C. During this incubation phase, THP-1 (1×10^6^ cells/ml) were loaded with 5 µM Calcein-AM in SBS buffer plus 0.01% (w/v) pluronic acid for 1 h at 37°C. Following loading, THP-1 cells were re-suspended to 10^6^ cells/ml in SBS buffer and primed with CCL2 (50 ng/ml) or vehicle (SBS buffer) for 45 min at 37°C. THP-1 cells were washed once before addition to washed HUVEC monolayers, allowing cells to adhere for 1 h at 37°C in a humidified 5% CO_2_ incubator. Non-adhered cells were aspirated and adhered cells were washed twice with SBS buffer before reading the fluorescence intensity (496 nm excitation; 516 nm emission).

### Luciferase–luciferin assay for nucleotide release

Release of nucleotides from THP-1 cells was assayed by means of an ATP bioluminescence assay kit (HSII kit, Roche, UK). THP-1 cells (10^6^ cells/ml in SBS buffer) were challenged with CCL2 (50 ng/ml) at room temperature prior to sampling (60 µl). Cells were immediately sedimented at 4°C and 20,000 ***g*** and clarified samples were mixed 1:1 with luciferase reagent before luminescence measurements were made using a Modulus Luminometer (Turner BioSystems, USA) with a 7-s integration time. Experiments were performed at room temperature (22–25°C).

### Ion-pair reverse-phase HPLC for nucleotide release

HPLC detection of nucleotides was performed using a Supercosil LC-18-T column (Sigma-Aldrich, UK) equilibrated with 10 column volumes of Buffer B (organic phase) and 30 volumes of Buffer A (mobile phase). Buffer A consisted of 39 mM K_2_HPO_4_, 26 mM KH_2_PO_4_ and 4 mM tetrabutylammonium hydrogen sulfate, pH 6.0. Buffer B consisted of 39 mM K_2_HPO_4_, 26 mM KH_2_PO_4_ and 25% (v/v) methanol, pH 6.0. Buffers were prepared in deionized water and filtered through a 0.4 µm filter. THP-1 cells (10^6^ cells/ml in SBS buffer), were challenged with CCL2 (50 ng/ml) at room temperature prior to sampling (200 µl). Cells were immediately sedimented at 4°C and 20,000 ***g***. Clarified samples were injected after two blank injections, and were compared with nucleotide standards.

### P2Y_6_-knockdown THP-1 cells

Lentiviral contructs using the pLKO.1-puro shRNA expression vector targeting the human P2Y_6_ receptor were obtained from the Sigma MISSION shRNA library. THP-1 cells (5×10^4^ cells/ml) were infected with the lentiviral knockdown vectors (MOI 10) or non-target control vectors for 72 h at 37°C in a humidified 5% CO_2_ incubator. To generate cells stably deficient in P2Y_6_, cells were selected with puromycin (1 µg/ml) for 4 days. All cultures were maintained in THP-1 culture medium as described above, supplemented with 1 µg/ml puromycin. P2Y_6_ mRNA transcripts were quantified in THP-1 and knockdown cells using SYBR Green-based quantitative PCR and the following primers: (sense) 5′-GCTCTCACTGTCATCGGCTT and (antisense) TCTGCCATTTGGCTGTGAGT-3′. Clone 5 stable knockdown was generated with the following shRNA sequence: 5′-CCGGTGGTCCGCTTCCTCTTCTATGCTCGAGCATAGAAGAGGAAGCGGACCATTTTTG-3′.

### Flow cytometry

10^5^ THP-1, P2Y_6_-knockdown or scrambled control THP-1 cells were washed with PBS before being labelled with a final concentration of 0.5 ng/ml phycoerythrin-conjugated anti-human-CCR2 or -mouse-IgG2a K isotype control antibodies (Biolegend). Cells were labelled in PBS with 1% (w/v) BSA for 30 min at room temperature. For antagonist experiments, THP-1 cells were pre-incubated with apyrase (2 U/ml), MRS2578 (1 µM) or appropriate vehicle for 30 min prior to labelling. Cells were washed twice in PBS and analysed on a BD Accuri C6. Gating excluded debris, and at least 10,000 cells were analysed for each experiment. FL2A represents fluorescence detection using a 585/25 band-pass filter and excitation at 488 nm.

### Statistical analysis

Data were analysed using Origin Pro 9.0 software (Origin Lab, USA). Concentration–response curves were fitted assuming a Hill coefficient of 1. Hypothesis testing for experiments with paired datasets was performed by means of paired Student's *t*-test, and by ANOVA for experiments with multiple datasets. Data are expressed as mean±s.e.m. of at least three independent experiments.

## Supplementary Material

Supplementary Material
