# Supplementary material for: P2Y6 receptor inhibition perturbs CCL2-evoked signalling in human monocytic and peripheral blood mononuclear cells
Source: J Cell Sci. 2014 Nov 15;127(22):4964–73. doi: 10.1242/jcs.159012 (PMC4231309; doi:10.1242/jcs.159012)
Supplement: Supplementary Material [file supp_127_22_4964__index.html]

P2Y6 receptor inhibition perturbs CCL2-evoked signalling in human monocytic and peripheral blood mononuclear cells — Supplementary Material 

# P2Y6 receptor inhibition perturbs CCL2-evoked signalling in human monocytic and peripheral blood mononuclear cells

## JCS159012 Supplementary Material

**Files in this Data Supplement:**

- **Supplementary Material**
