# Supplementary material for: P2Y6 receptor inhibition perturbs CCL2-evoked signalling in human monocytic and peripheral blood mononuclear cells
Source: J Cell Sci. 2014 Nov 15;127(22):4964–73. doi: 10.1242/jcs.159012 (PMC4231309; doi:10.1242/jcs.159012)
Supplement: Supplementary Material [file supp_127.22.4964_JCS159012.pdf]

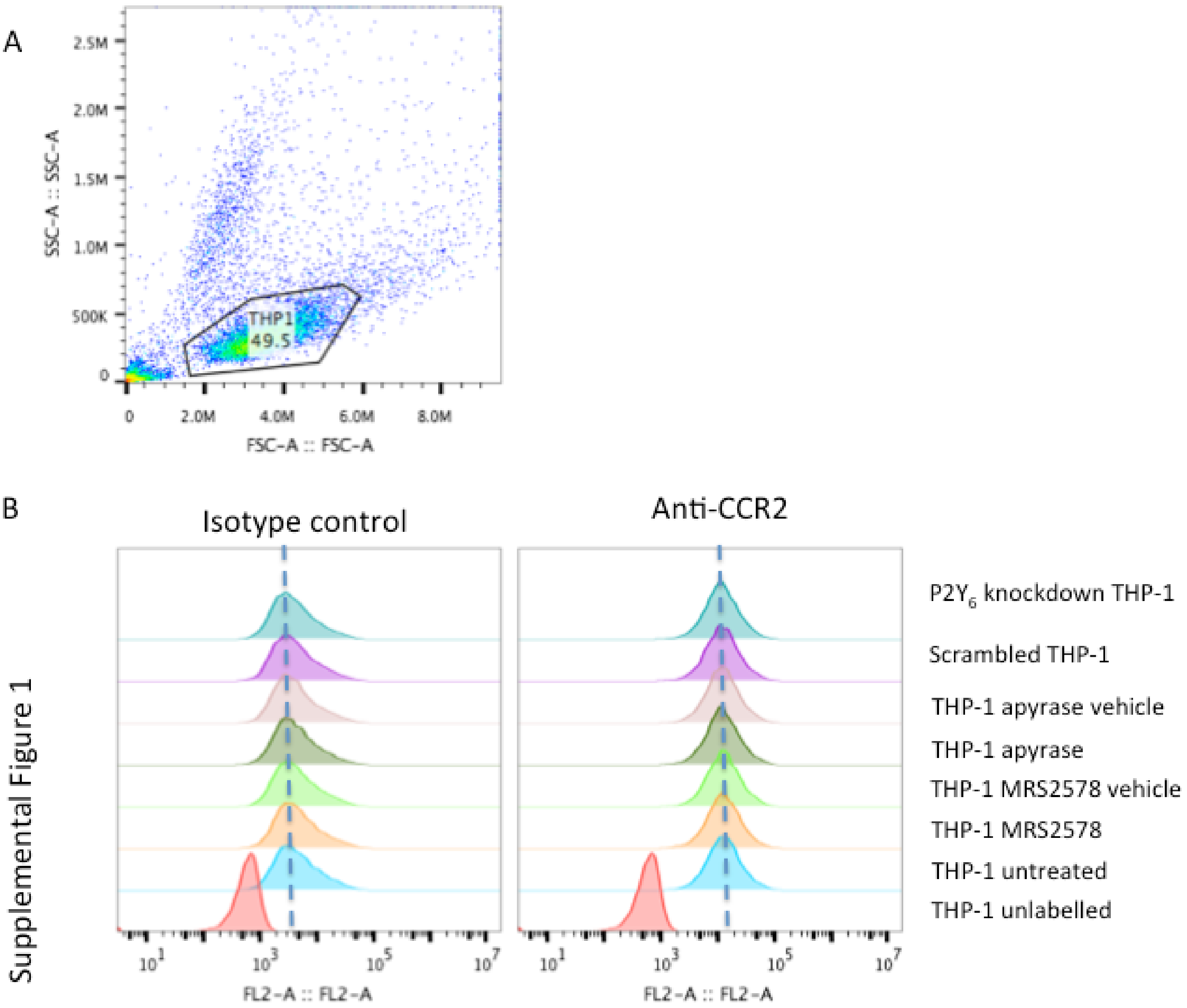

Fig. S1 Analysis of cell surface CCR2 expression by flow cytometry. (A) Representative FSC:SSC plot showing gating selection of unlabelled viable THP-1 cells. (B) Half-offset histogram overlays for isotype control or anti-CCR2 labelled for scrambled versus $P2Y_6$ knockdown, and for THP-1 cells pre-incubated with apyrase (2 U/mL; 30 mins), MRS2578 (1mM; 30 mins) or vehicle control.
